# Potential role of a novel dual energy lattice-tip catheter in re-ablation of recurrent premature ventricular contractions: a case series

**DOI:** 10.1007/s10840-026-02342-6

**Published:** 2026-05-02

**Authors:** Laura Rottner, Christian Gold, David Leistner, Reza Wakili

**Affiliations:** 1 University Heart and Vascular Center Frankfurt, Goethe University Frankfurt, Frankfurt am Main, Germany; 2 German Center for Cardiovascular Research DZHK, partner site Rhine-Main, Mainz, Germany; 3 University Heart and Vascular Center Frankfurt, Theodor-Stern Kai 7, Frankfurt am Main, 60596 Germany

In ventricular premature contraction (PVC) ablation, precise mapping, localization of the ectopy origin as well as achievement of transmural and durable lesions is critical in ensuring procedural success. However, it remains challenging in some cases, especially when the substrate is localized in certain regions, for instance, in the epicardium or the left ventruclar (LV) summit [1]. Recent advancements include pulsed-field ablation (PFA), in combination with or without radiofrequency ablation (RFA), as well as innovative catheter designs that offer a larger effective surface area. Among these promising technologies is the Affera™ mapping and ablation system.

The effectiveness and safety of this system have been previously clinically validated for the treatment of atrial arrhythmias [2]. The potential application of the system for lesion creation in ventricular myocardium has been studied preclinically [3]. However, so far, clinical experience applying this novel system for ablation in the ventricle is scarce. Data from a multicenter registry as well as smaller case series evaluating its use within the ventricle are promising [4, 5]. However, there are still concerns about safety for its application near to coronary arteries, as some rare cases of (subclinical) spasm are reported [5].

With the present case series, we aim to share our initial experience with the use of Affera™ and the Sphere-9™ catheter for the re-ablation of different types of PVCs after an initial non-successful ablation attempt.

This analysis was approved by the institutional review board and the local ethics committee (Frankfurt Arrhythmia registry, FRAME: registration number: 2023 − 1379).

The Affera^™^ mapping and ablation system has been described in detail previously [2]. The procedures were performed under deep sedation. After vascular access, a diagnostic catheter was introduced via the right femoral vein and positioned within the coronary sinus. Depending on the supected target location the left ventricle (LV) was accessed either transseptally or retrogradely, if left-sided origin was assumed. Activation and pace mapping were used to identify target region. The primary ablation strategy was RFA (temperature control mode with a target temperature of 60 °C and 40 Watts current limit with 30 s application duration) in order to maintain detailed signal-based mapping as long as possible. If RF ablation alone could not achieve acute PVC suppression after careful mapping or teh operator decided to consolidate lesions, PF applications (1500 pulses/train, 12 trains/application, 350 ms inter-train delay, 5 s application duration) were added. No nitrates were administered prophylactically before PFA. 12-lead holter ECG was performed the day after the procedure in all patients before discharge and all patients were seen in the outpatient clinic 3 months after ablation to judge for ablation success.

A total of 10 consecutive patients (aged 66 ± 14, 3/10 (30%) women, 3/10 (30%) with structural heart disease) with symptomatic PVC undergoing re-do ablation procedures using a novel mapping and ablation system (Affera^™^, Medtronic Inc.) were analysed. 3/10 (30%) of all patients already underwent ≥ 2 previously failed RFA procedures.

The mean procedure duration was 100 ± 35 min, with a mean fluoroscopy time and dosage of 12 ± 7 min and 11 ± 7 Gycm^2^. The arrhythmogenic focus was identified within the right ventricular outflow tract (RVOT) in 3/10 (30%), within the LV summit in 4/10 (40%), at the mitral annulus in 2/10 (20%) and within the aortic cusp in 1/10 (10%) patients. On average, 7 ± 4 RF and 5 ± 5 PF applications were delivered per patient. In 3/10 (30%) patients only RFA was performed. In 3/10 (30%) patients additional PFA was needed to achieve acute PVC suppression and in 4/10 (30%) patients the operator decided for prophylactic lesion stacking via additional PFA although RF only already led to acute PVC suppression. There was no patient, in whom only PF was applied.

The ablation resulted in sustained PVC suppression in all patients with no recurrence after stop of sedation and a 30-minutes waiting time. No periprocedural complications were documented. Also, in those cases with use of PF energy in proximity to coronary arteries, no ECG changes attributable to ischemia were noted. All patients were discharged two days after the procedure without symptoms. 2/10 patients (20%, 1x PVC from anterior mitral annulus and 1x PVC from LV summit) showed symptomatic PVC recurrence within short term follow-up (mean follow-up 97 ± 54 days). However, significant PVC burden reduction was achievied in all patients (see central illustration). Of note, one patient died for unknown reason 2 months after the procedure.

The main findings of this study are as follows: (1) mapping and ablation of PVCs using a large-footprint catheter, enabling both RFA and PFA, is feasible and (2) associated with promising acute and short-term effectivness, also for challenging regions (i.g. LV summit or mitral annulus), where conventional systems and tip catheter designs bear limitations, (3) lesion stacking using RFA followed by PFA is necessary and advisable to achieve longterm PVC suppression, and (4) RF- as well as PF-application was safe in our study and ablation using the lattice-tip catheter in proximity to coronary arteries did not induce clinical spasm.

Further data on optimal ablation strategy (RF followed by PF vs. RF or PF only), the relevance of (subclinical) coronary spasm depending on ablation region are desirable and long-term efficacy in larger cohorts crucial to finally judge for future areas of applications.

**Central Illustration**

**Figure:**
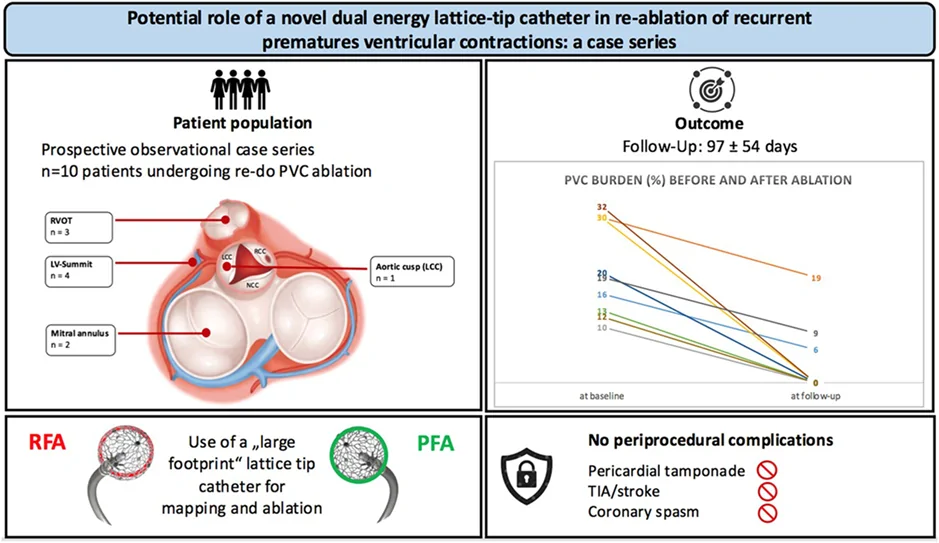

LV= left ventricular; RVOT= right ventricular outflow tract; RFA= radiofrequency ablation; PFA= pulsed-field ablation; LCC= left coronary cusp; PVC= premature ventricular contractions; TIA= transient ischemic attack.

## Data Availability

No datasets were generated or analysed during the current study.
